# Supplementary material for: Feature selection for high dimensional microarray gene expression data via weighted signal to noise ratio
Source: PLoS One. 2023 Apr 25;18(4):e0284619. doi: 10.1371/journal.pone.0284619 (PMC10128961; doi:10.1371/journal.pone.0284619)
Supplement: Attachment — Submitted filename: Response to Reviewers.pdf [file pone.0284619.s001.pdf]

Dear Editor,

Thank you for allowing us to update our manuscript to be considered in your prestigious journal. The point-to-point response to the reviewers' comments is as follows:

Reviewer # 1:

Comment:
The overall impression of the technical contribution of the current study is reasonable. However, the Authors may consider doing necessary amendments to the manuscript for better comprehensibility of the study.

Response:

Thank you for your feedback. The whole manuscript is revised and all the necessary amendments have been made. We hope the manuscript is now in better position in terms of comprehensibility.

Reviewer # 2:

Comments
Abstract must be rewritten with objectives included and mention accuracy rate of performance. Write the problem statement.

Response:

Thank you for your concern. The abstract is now revised by including objectives and problem statement. Moreover, the performance is evaluated on the basis of error rates, and one can easily determine the accuracy rates of the selected genes, as it is a complement of classification error rate.

Comment # 2: Mention the contributions made in the paper.

Response:
Thank you for the suggestion. The contribution made in the paper is mentioned by updating the abstract and introduction sections.

Comment # 3:
Discuss the Related work.

Response:
Related work is now thoroughly discussed.

Response:

Thank you for your suggestion. The related work is further updated by including some recent and related literature in the field of feature/gene selection in high dimensional gene expression datasets.

Comment # 5:
Justify your approach with the help of performance parameters.

Response:

Thank you for the suggestion. The method ranks features based on weighted signal to noise ratio and selects the top ranked features. We do not associate any hyper parameter of the method.

Comment # 6: Include Table of Comparison with the state-of-the-art literature.

Response:

Thank you for your suggestion. The Table of comparison is further updated by including a new feature selection method i.e. (SVM-mRMRe). The results of this new method are updated throughout manuscript.

Comment # 7: Proposed method (WSV − Sn) needs to be defined and called it with a precise name.

Response:

Thank you for your suggestion. The proposed method is redefined. Moreover, the name (WSV − Sn) is now replaced with precise name.

Reviewer # 3:

Comment # 1: The abstract is not attractive. Some sentences in abstract should be modified to make it more attractive for readers. In addition, the repetition should be removed (see the word 'the proposed method.

Response:

Thank you for your concern. The manuscript is updated by doing the required changes.

Comment # 2: In Introduction section, it is difficult to understand the novelty of the presented research work. This section should be modified carefully. In addition, the main contribution should be presented in the form of bullets.

Response:

Thank you for identifying this flaw. The manuscript is revised by including novelty of the proposed research work. Moreover, the main contribution is now presented in the form of bullets.

Comment # 3: The authors should discuss the existing work properly. In addition, the following works should be discussed: A smart healthcare monitoring system for heart disease prediction based on ensemble deep learning and feature fusion, An intelligent healthcare monitoring framework using wearable sensors and social networking data, Artificial intelligence in disease diagnosis: a systematic literature review, synthesizing framework and future research agenda, A tri-stage wrapper-filter feature selection framework for disease classification.

Response:

Thank you for your concern. The manuscript is updated by discussing the related work properly. Moreover, the studies suggested by your good self are also incorporated.

Comment # 4: Figures are blurred, their quality should be improved.

Response: Thank you for the suggestions. The quality of figures in the manuscript is now improved.
Comment # 5: Captions of the Figures not self-explanatory. The caption of figures should be self-explanatory, and clearly explaining the figure. Extend the description of the mentioned figures to make them self-explanatory.

Response: Thank you for the suggestion. The manuscript is updated by changing the caption of all figures. The caption are now self-explanatory.

Comment # 6: More details should be included in future work.

Response: Thank you for the suggestion. The required changes have been made and highlighted in the manuscript.

Comment # 7:The whole manuscript should be thoroughly revised in order to improve its English**.**

Response: Thank you for your feedback . The whole manuscript is revised by improving its language.
